# Early injury responses to nutrient treatment in adult *Drosophila* limb

**DOI:** 10.1242/bio.062737

**Published:** 2026-10-23

**Authors:** Yutian Li, Jesus del Rio Salgado, Gloria Bates, Zevin J. Condiotte, Maya Caskey, Christian M. Dimayuga, Brian Williams, Ingileif Hallgrimsdottir, Lior Pachter, Lea Goentoro

**Affiliations:** ^1^ Division of Biology and Biological Engineering, California Institute of Technology, Pasadena, CA 91125, USA; ^2^ Department of Computing and Mathematical Sciences, California Institute of Technology, Pasadena, CA 91125, USA

**Keywords:** *Drosophila*, Nutrients, Injury

## Abstract

It was previously observed that partial regrowth can be induced to begin in adult *Drosophila* limb by modulating nutrients. In this work, to further investigate this observation, we characterized the early cellular- and tissue-level processes that are promoted by nutrient treatment after injury. We find that response to nutrient treatment can be readily identified within hours, by alteration in wound healing. Subsequently, at day scale, dramatic reversal of large-scale tissue loss occurs in treated flies. Finally, at week scale, tissue remodeling begins, and a dome-shaped protrusion forms at the injury site. Using single-nucleus RNA sequencing at this stage, we find widespread transcriptional modulations across cell types. Distinct muscle and epithelial cell types emerge that are enriched in mitotic and morphogenetic genes, including those associated with regeneration in larval tissues. These tissue-, cell-, and gene-level processes characterized here provide a framework for the previously observed partial limb growth and for further investigating the suggestion of regeneration induction in adult *Drosophila* through nutrient treatment.

## Teaser

**Summary:** Adult fruit flies do not normally regrow limbs, but nutrient treatment can induce partial regrowth. This study characterizes the early tissue, cellular, and gene expression changes after injury promoted by nutrient treatment.

## INTRODUCTION

Although some animals have a remarkable ability to regenerate, some others have more limited regenerative capacity. To address this question, building on insights from highly regenerating animals (Tanaka and Reddien, 2011; Sánchez Alvarado, 2000; Poss and Tanaka, 2024), an increasing number of studies have begun to examine poorly regenerating systems and ask how, or whether, regeneration can be induced. Excitingly, evidence that regeneration processes can be induced is emerging. For instance, in frogs, limb regeneration can be induced by activating the Wnt pathway (Kawakami et al., 2006), injecting limb progenitor cells (Lin et al., 2013), or administering a cocktail of drugs (Murugan et al., 2022). In adult mice, digit bone regrowth can be induced by administering Bmp to the digit stump (Yu et al., 2019), and heart regeneration can be promoted by hypoxia (Nakada et al., 2017) or by activating the Hippo pathway (Xin et al., 2013). While the induction is not complete, these studies demonstrate that regeneration processes can be promoted and provide experimental frameworks to further identify factors that limit regeneration.

While regeneration induction has been mostly studied in frogs and mice, recent studies suggest that regeneration processes can potentially be induced in the adult fruit fly, *Drosophila melanogaster* (Abrams et al., 2021). *Drosophila*, along with beetles and butterflies, belong to the vast group of holometabolous insects that do not regenerate adult limbs (Worley et al., 2012). This is in contrast to the hemimetabolous insects that regrow limbs during molting at nymphal stages (Khan et al., 2016). It was reported in a previous study that feeding specific nutrients after amputation produced partial limb regrowth in adult *Drosophila* (Abrams et al., 2021; reproduced in Fig. 1; Bates et al., in review, 2026; and Li et al., in review, 2026). Moreover, nutrient-dependent effects on appendage regrowth were not only reported in *Drosophila* but also observed in multiple species, from jellyfish to frogs to mice (Abrams et al., 2021; Williams et al., 2021). The role of nutrients brings energy consideration into the problem of promoting tissue regrowth and supports the hypothesis that energy and resources may have been factors in the evolution of regeneration.

If verified further, regeneration induction in *Drosophila*, with its rich experimental toolbox, small size, and shorter life cycle, can be a powerful phenotype to facilitate the identification of key principles to test in vertebrate systems. Motivated by the potential use of *Drosophila*, while the reported partial limb regrowth is at month scale, we sought in this study to characterize the early responses to the nutrient treatment. This work does not address the mechanisms of the reported month-scale regrowth (e.g. whether it proceeds through epimorphic regeneration, hyperplasia, or other processes). However, the day- and week-scale gene, cellular, and tissue markers reported here may facilitate the investigation of these more mechanistic questions.

## RESULTS

### Nutrient treatment rapidly alters wound healing

Amputation of the limb and treatment were performed as described in Fig. 2A,B. In control flies, the wound heals similar to what has been described in wound studies in larvae (Galko and Krasnow, 2004; Bidla et al., 2005; Álvarez-Fernández et al., 2015). Within minutes, hemolymph flow stops. Within 12–72 h, a thick, melanized scab forms over the wound site (Fig. 2C,D). We previously called this phenotype ‘clotting’ (Abrams et al., 2021). However, hemolymph flow still stops within minutes, consistent with the previous report that soft-clot formation is independent from the subsequent hardening of the clot into a scab (Bidla et al., 2005). We therefore use ‘scabbed’ and ‘scabless’ to describe the presence or absence of the visible melanized scab. Interestingly, we also noticed that a small fraction of flies (median 8.6%) heals wounds without forming a melanized scab (e.g. Fig. 2E,F). We did not notice this low baseline scabless healing in our previous work (Abrams et al., 2021), likely reflecting the larger sample size in this study and the more consistent gentler handling of the flies in this study (as described in the Materials and Methods).

**Fig. 1.**
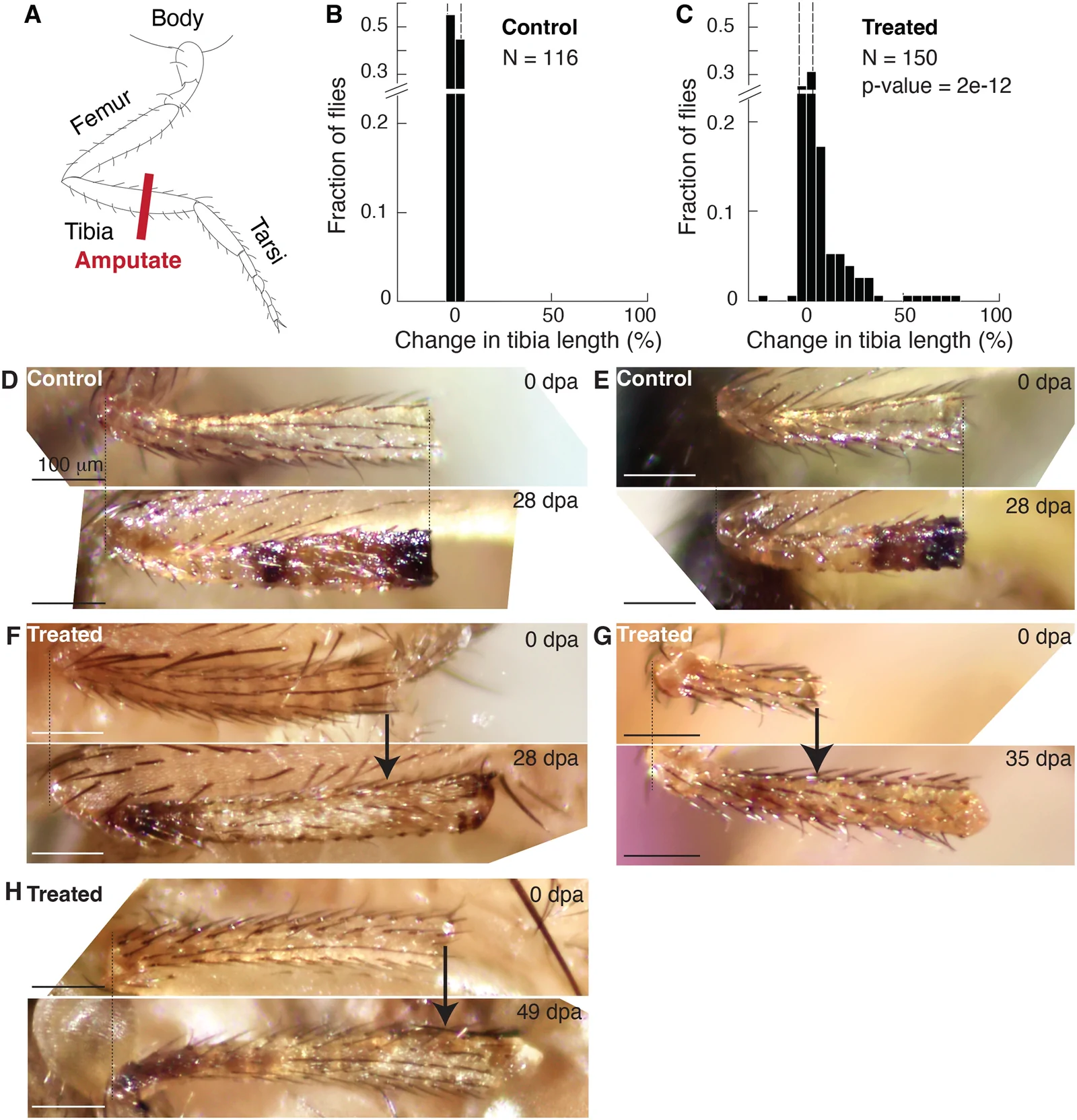
**Adult fruit flies do not normally regenerate limbs.** It was previously observed that providing specific nutrient factors can promote partial limb regrowth. (A–C) These data are reproduced from Abrams et al. (2021). (A) In these experiments, fly limbs were amputated across the tibia segment (more experimental details are presented in Fig. 2). Treated flies were supplemented with LGI. (B,C) Length of the tibia stump was measured immediately after amputation and then 3–4 weeks later. Plotted here is the change in the tibia length in control (B) and treated flies (C). Dashed lines are the 95% confidence intervals (CIs) of the control distribution. 49% of the tibias from treated flies show growth beyond the control 95% CI. (D,E) Examples of control limbs. No growth occurs. (F) An example of a treated limb that shows partial regrowth. This is an independent replication of Abrams et al.’s (2021) findings. (G,H) More independent replications of partial regrowth. Consistent with the role of nutrients, partial regrowth can be promoted by nutrient-related factors, i.e. microbiome modulation (G; reproduced from Bates et al., in review, 2026) and the steroid hormone ecdysone (H; reproduced from Li et al., in review, 2026). Scale bars: 100 µm (D–H).

**Fig. 2.**
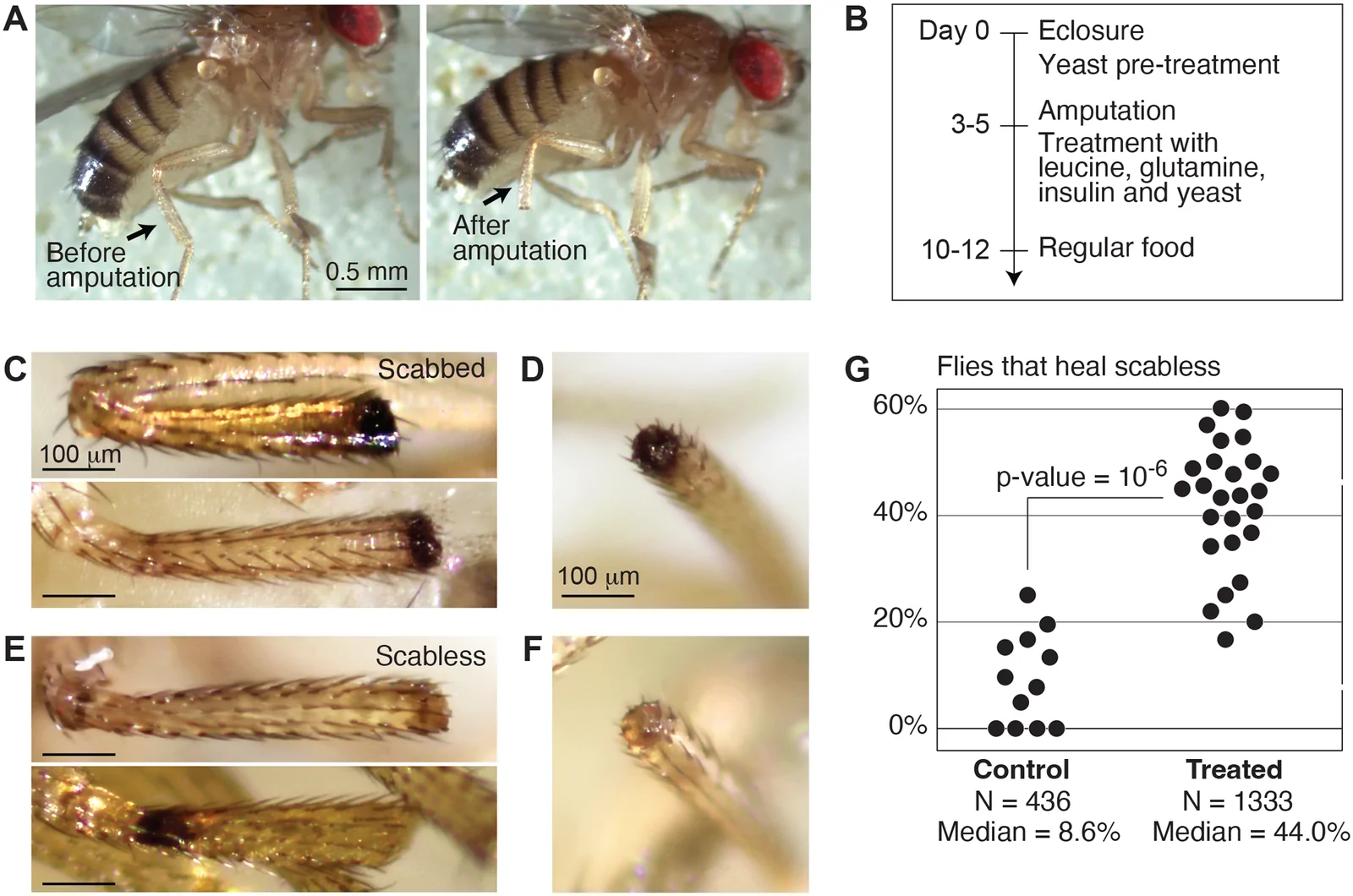
**Treatment alters the way the wound heals.** (A) Flies were gently anesthetized and amputated across the tibia segment. Shown is a fly before (left) and after (right) amputation. (B) In all experiments performed in this study, for treatment, newly eclosed flies were placed on food supplemented with yeast for 3–5 days and then amputated. After amputation, flies were placed in food supplemented with LGI and yeast for a week (see Materials and Methods for more details), after which they were moved to regular food. Control flies remain in regular lab food the entire time. (C,D) Flies normally heal wounds by forming a melanized scab. (E,F) Flies that heal wounds without a melanized scab. Scale bars: 100 µm (C–F). (G) Each data point is an independent experiment, with 30–40 flies examined in each experiment. *N*: the total number of flies analyzed. Statistical test: Kruskal–Wallis.

The scabless healing is interesting because the treatment significantly increases the fraction of flies that heal scabless (to a median of 44%, Fig. 2G). Melanization may occur in proximal regions (e.g. Fig. 2E, bottom) or the distal tissue may melanize darker later on (e.g. Fig. 5A). The frequency of scabless healing, baseline and treated, can vary across experiments. Since the treatment involves nutrient signaling, underlying variations in metabolic states (e.g. Pettersen et al., 2018) may play a role. In addition, other factors such as genotype (Table S1) as well as stress (Li et al., 2023b) can also influence wound healing. While we do not fully understand the basis of the variation, we verified that the treatment reproducibly increases the fraction of flies that heal scabless (Fig. S1).

In summary, nutrient treatment readily alters wound healing, which can be assessed within hours after amputation. The nutrient treatment appears to allow what is normally a marginal response in the population to emerge in many more flies. As described next, the way the wound heals correlates with subsequent tissue processes at day scale.

### Nutrient treatment prevents large-scale tissue loss

To characterize tissues, we first attempted staining. Because of the thick adult cuticle, present histological protocols involve chemical clearing and shaking incubation over multiple days. While these protocols work for intact tissues, the extensive handling damages tissues in the scabless limbs. While we continue developing staining strategies, in this study, we utilized fluorescent reporters.

To determine the reporter lines to use, we first assessed the technical aspects of using transgenic lines. First, genetic background may influence response to treatment. We screened multiple fluorescent reporter lines and used lines that are most responsive to treatment (Table S1). Next, fluorescence imaging is often hampered by the thick cuticle. Fortunately, a recent study (Koyama et al., 2020) examined how mounting in Elmer's glue optically clears the adult cuticles. With the glue mounting, the reporter lines used here show a strong signal-to-noise ratio and can be readily imaged in freshly dissected limbs (Fig. S2). Finally, autofluorescence is a marker of tissues with increased metabolic activities (e.g. Monici, 2005; Morrow et al., 2024), which can mark response to injury. Autofluorescence is indeed slightly elevated in the injured limbs, but the low, diffuse autofluorescence is distinguishable from the strong, structured signal from the reporter lines used in this study (Fig. S3).

To visualize tissues in the limb, we chose to focus on the Myocyte enhancer factor 2 (Mef2)>red fluorescent protein (RFP) line, which expresses RFP driven by the promoter of *Mef2*. Mef2 is a well-established regulator of muscle differentiation and maintenance, and it is widely used as a muscle marker in *Drosophila* (e.g. Black and Olson, 1998; Soler et al., 2012; Taylor and Hughes, 2017). Mef2 has also been found to function in diverse cell types (Potthoff and Olson, 2007; Taylor and Hughes, 2017). We chose to focus on the Mef2>RFP line because it is the most responsive line in our screen (Table S1), it shows a strong signal-to-noise ratio (Fig. S2), and it labels a major tissue component of the adult limb. Consistent with its dominant role in muscle and muscle being a prominent tissue in the limb, Mef2>RFP flies show strong striated muscle patterns in the limb (Fig. S2). Thus, Mef2>RFP provides a clear readout for tissues.

Having examined the technical aspects, we analyzed Mef2>RFP signals after amputation. As alluded earlier, tissue responses correlate with the way the limbs heal. In limbs that heal scabbed, from control or treated flies, Mef2>RFP signals gradually diminish over days and disappear within 12 days after amputation (Fig. 3A,B,D; Fig. S4). The loss of tissue signals is largely contained within the injured leg segment (the tibia); femur signals are occasionally also affected. Tissue loss in major injury is not unexpected. It is also a major problem in human amputees (Finco et al., 2022).

**Fig. 3.**
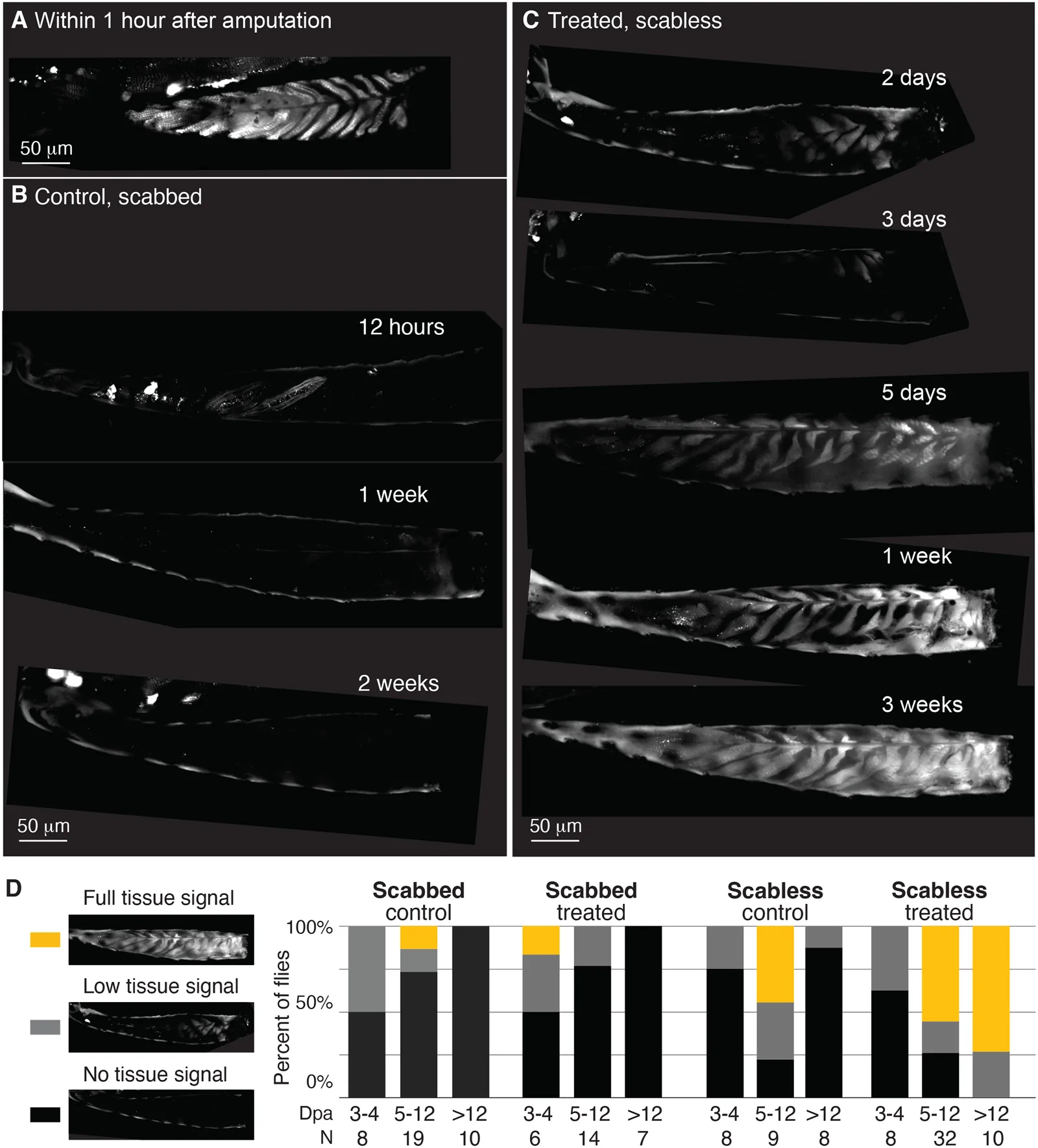
**Tibia tissues survive in treated flies that heal scabless.** Mef2>RFP flies were amputated and treated as described in Fig. 2B. To image, the limbs were dissected at the indicated time after amputation, mounted, and imaged using a confocal microscope. Each image is a representative tibia from an independent fly at the indicated time point. (A) A tibia within 1 h after amputation (shown magnified in Fig. 4A). (B) Representative tibias from control flies that heal scabbed. (C) Representative tibias from treated flies that heal scabless. (D) Summary of the samples analyzed. Black, no tissue signal. Gray, low tissue signal. Yellow, full tissue signal.

**Fig. 4.**
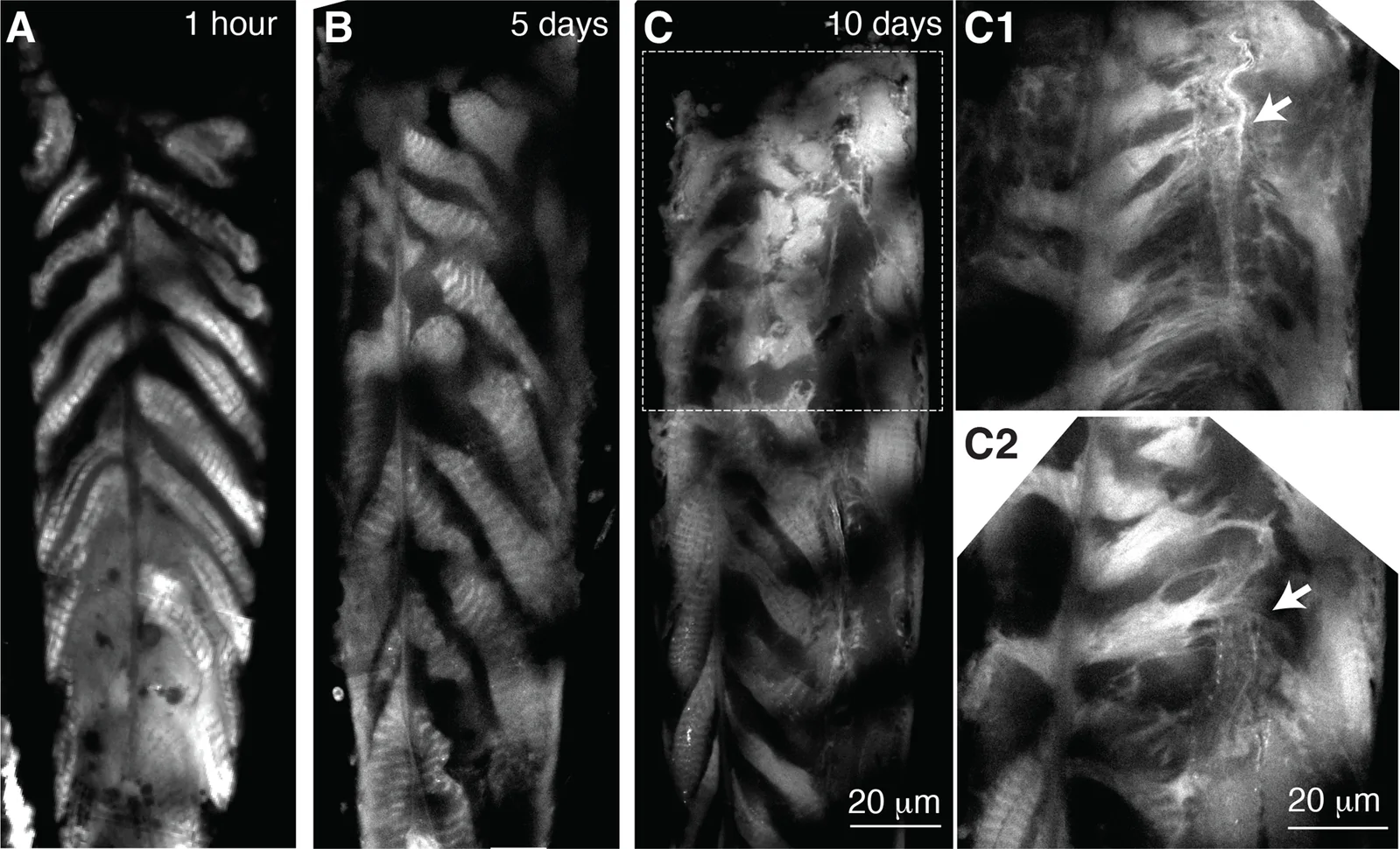
**Around 7–10 dpa, tissues near the amputation plane began showing morphological changes.** In these experiments, Mef2>RFP flies were dissected at the indicated time after amputation and imaged using confocal microscopy. A is a magnified view of the same image shown in Fig. 3A. Figures C1 and C2 are a magnification of the boxed region in C, taken at *z*-sections deeper into the tissues.

In limbs that heal scabless, from control or treated flies, Mef2>RFP shows dynamic expression. After amputation, the Mef2>RFP signal is at first reduced within 3–4 days after amputation. Afterward, the Mef2>RFP signal returns (Fig. 3C,D). In control limbs that spontaneously heal scabless, the return of the tissue signals is not sustained, disappearing by 12 days (see scabless control data in Fig. 3D). By contrast, in treated limbs that heal scabless, the treatment not only significantly increases the frequency of flies that heal scabless (Fig. 2G), it also sustains tissue survival to multiple weeks (Fig. 3C,D). This suggests that both scabless healing and treatment are necessary for sustained tissue survival.

To further verify tissue survival, we analyzed another reporter, NRE-EGFP. NRE-EGFP is a reporter for Notch signaling (Zacharioudaki and Bray, 2014), a known process in tissue repair (Bi et al., 2016). We observed Notch reporter activation in the treated, scabless limbs (Fig. S5). The Mef2>RFP and NRE-GFP analysis signals confirm survival of limb tissues. It should be noted that the analyses here rely on reporter lines; thus, further molecular analysis is needed to confirm the specific molecules involved.

In summary, within days after amputation, nutrient treatment reverses large-scale tissue loss. While tibia tissues in flies that heal scabbed degenerate, tibia tissues in flies that heal scabless survive, and the treatment is required to increase the proportion of flies that heal scabless and sustain tissue survival to week scale. Finally, to further verify that the scabless healing and treatment are meaningfully necessary for the subsequent tissue processes described next, we verified that growth occurs only in the treated scabless limbs (Fig. S4).

### Morphological changes begin at 1 week onward

Next, we examined the Mef2>RFP signals in treated, scabless limbs more closely. Shown in Fig. 4A,B is a close-up of the Mef2>RFP signals hours after amputation (Fig. 4A) and 5 days later after the return (Fig. 4B). From ∼7 dpa onward, small muscle bundles begin appearing near the amputation plane (dashed box in Fig. 4C). Imaging deeper into the tissues (Fig. 4C1,C2) reveals that the small bundles are fraying at one end (e.g. see arrows). None of these bundles were apparent in limbs earlier than 7 dpa (Fig. 4A,B).

**Fig. 5.**
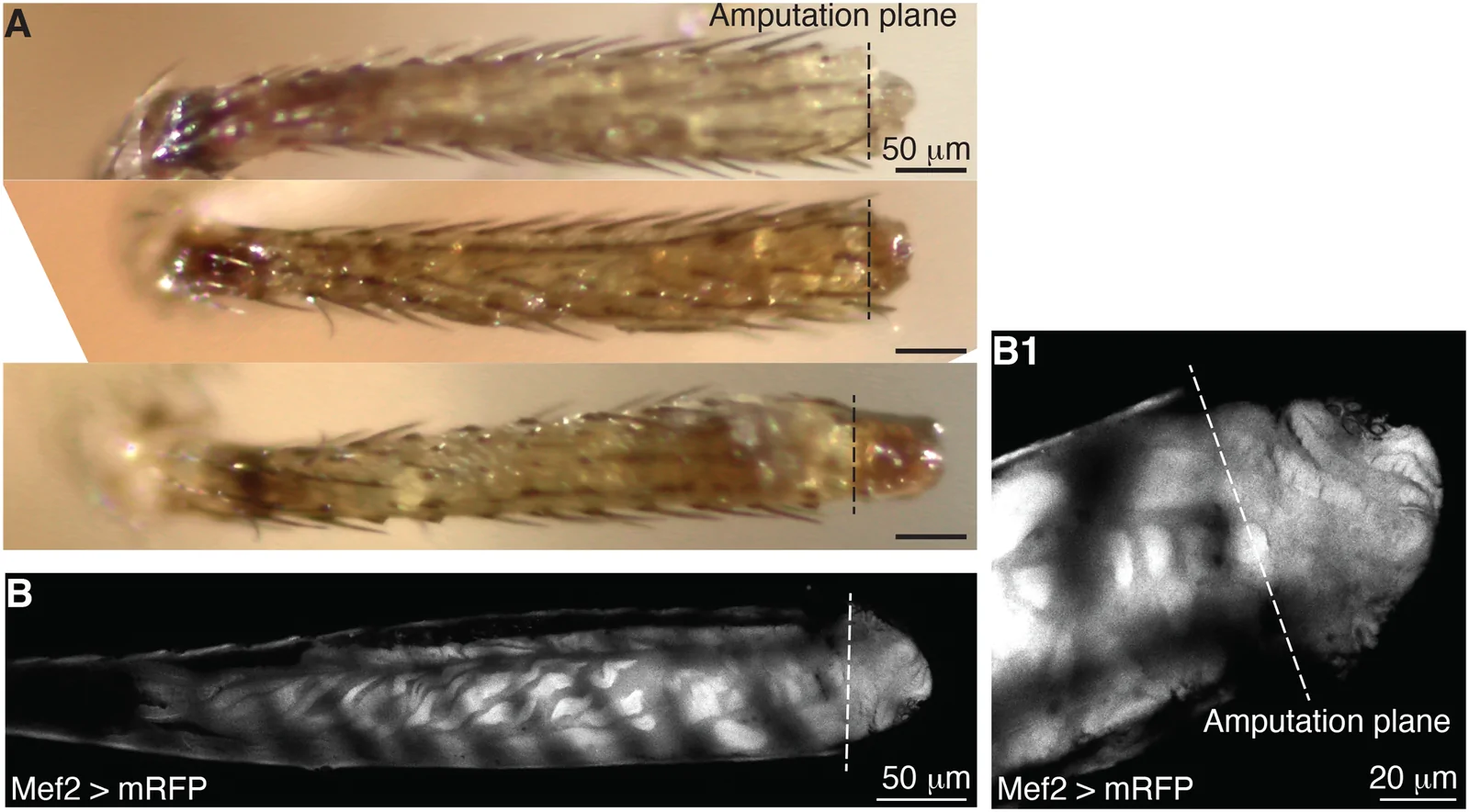
**Around 7–10 dpa, tissues begin growing over the amputation plane.** (A) Three different limbs, showing varying shapes of protrusion. (B,B1) The protrusion is positive for Mef2>RFP. B1 is a magnification of the distal region in B. The dashed line indicates the end of the cuticle.

Around the same time, tissue protrusion appears (Fig. 5). The onset of the protrusion varies from limb to limb. At any given time (we assayed at 7, 10, and 14 dpa), we would observe protrusion in ∼5–15% of the treated limbs (*N*=444). No protrusion was observed in treated flies that heal scabbed or in untreated flies. The shape of the protrusion ranges from a small dome to a long, cylindrical shape (Fig. 5A). In the Mef2>RFP assay, muscle bundles can be detected within the protrusion (Fig. 5B,B1).

In summary, morphological changes begin at week scale. Small muscle bundles appear near the amputation plane, and growth over the amputation plane begins. To resolve the cellular basis of the onset of these changes, in the next section, we describe the single-nucleus RNA sequencing analysis.

### Onset of morphological changes involves formation of new, large cell clusters not normally found in limbs

To facilitate future mechanistic studies, we next set out to characterize the cell types that are present in the surviving limb tissues. To characterize these surviving tissues at a stage most closely associated with potential growth, we focused specifically on when protrusion first begins to appear because protrusion formation provides the clearest visible indication of the onset of morphological changes (Fig. 5). To analyze cells in the adult, the Fly Cell Atlas (Li et al., 2022) relies on isolated single nuclei because recovering intact cells is difficult from cuticular body parts. Additionally, nuclear analysis is the only approach for multinucleated cells such as muscles. Accordingly, we performed single-nucleus RNA sequencing. To define cell types normally present in the limbs for comparison, we collected the same region of limbs from intact – uninjured and untreated – flies (Fig. 6A). Four replicates from four independent fly cohorts were collected. We analyzed a total of 27,859 nuclei from intact limbs (from four replicates: 4235, 6625, 9225, 7774 nuclei) and 32,635 nuclei from surviving injured limbs (from four replicates: 7340, 6877, 10,005, 8413 nuclei). For each nucleus, the average number of reads was ∼1×10^5^, and the average unique molecular identifier (UMI) count was ∼1×10^3^.

**Fig. 6.**
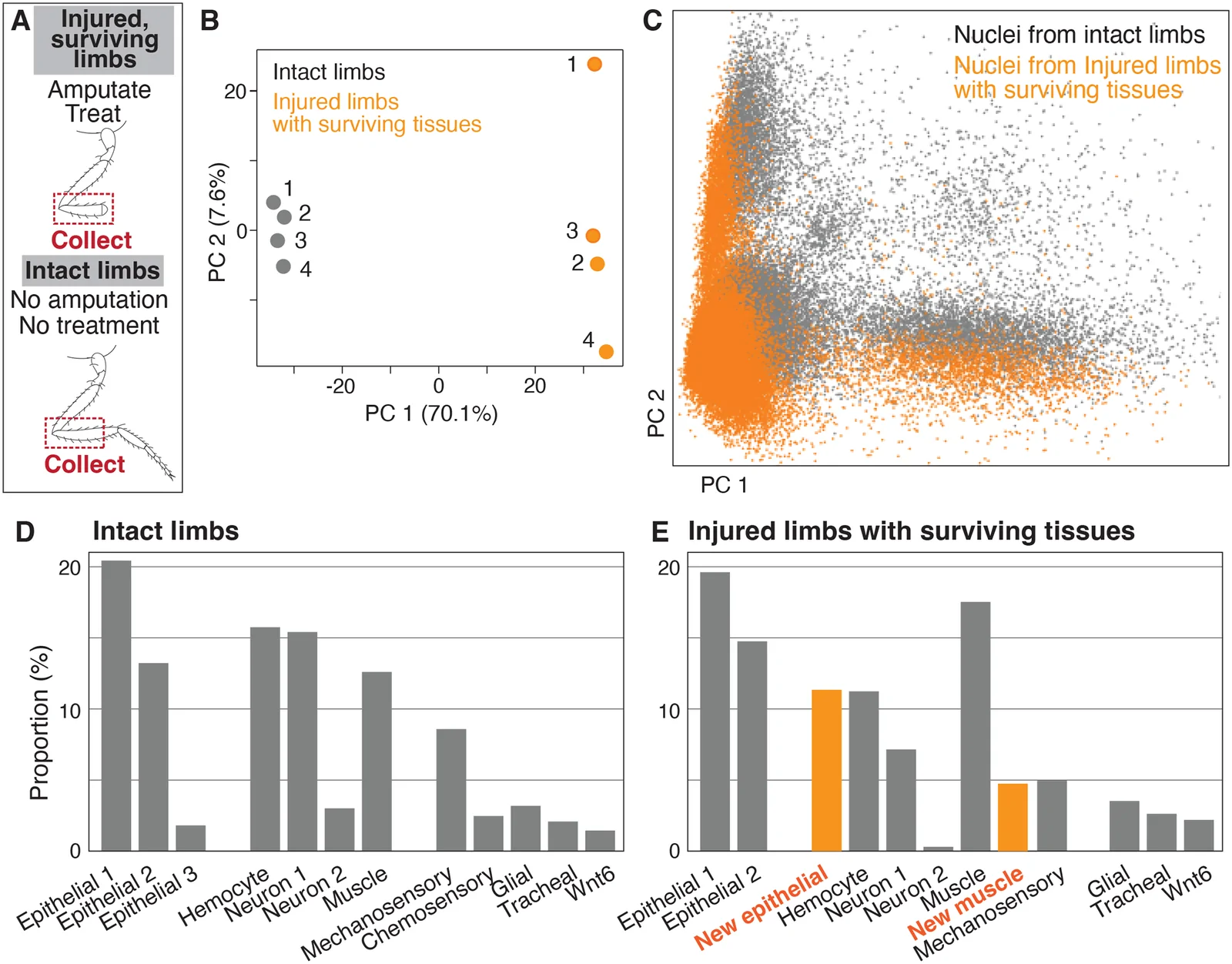
**Single-nucleus RNA-sequencing analysis recovers two distinct cell clusters in injured, surviving limbs.** (A) Sample collection design. We collected tibias at the protrusion stage (Fig. 5). The red dashed box indicates the region collected. To define normal-limb cell types for comparison, we collected the same region of tibias from uninjured, untreated flies. A minimal bit of femur had to be included to enable handling. Four replicates were collected, with each control-treated pair collected side by side from the same fly cohort. The four replications were performed in four different weeks to ensure we sampled independent fly cohorts. (B,C) PCA at the sample level (B) and nuclear level (C). (D,E) Cell clusters from intact limbs (D) and injured limbs with surviving tissues from the protrusion stage (E). Orange cell clusters are recovered only in injured, surviving limbs.

Using the single-nucleus data, we first performed principal component analysis (PCA; Fig. 6B). The samples clustered by condition, with PC1 capturing 70.1% of the variance. PC2, which correlates with technical batches, captures only 7% of the variance. This analysis verifies that the differences between the samples are predominantly biological, with minimal variations across technical replicates. Indeed, performing the PCA at the cell level, we verified the same population structures, but with a strong shift, between the samples, suggesting widespread transcriptional modulations between the two conditions (Fig. 6C). Top genes that are upregulated in the surviving injured limbs include multiple heat shock proteins (*Hsp23*, *Hsp68*, and *Hsp70*s) and genes in the JAK/STAT signaling pathway (*upd2* and *upd3*); both are pathways known to be involved in stress, immune, and repair processes. As we will analyze next, these genes are upregulated across multiple cell types.

To assess limb cell types, we performed clustering analysis. First, we verified that our analysis reproduces the Fly Cell Atlas (Fig. 6D). The normal limbs recover all major cell types in the limb: epithelial (marked by *grh*), muscle (*Mhc*, *sls*, *bt*), hemocyte (*Hml*), neuron (*fne*, *futsch*), tracheal (*trh*), and glial (*repo*). Not only does our analysis recover the marker gene separation, the top genes in each cluster reproduce those in the Fly Cell Atlas (Table S2). One cell type in the Fly Cell Atlas is missing in our dataset: the chordotonal organ, which is expected since the upper femur was not included in the sample. Moreover, with almost four times the number of nuclei than the limb atlas, the present analysis can further distinguish the following: (1) another population of epithelial cells marked by a lower expression of *grh* and whose top markers include *slf* and *knrl* (Epithelial 2, Fig. 6D); (2) following the marker genes identified in Hopkins et al. (2023), two populations of sensory support cells, mechanosensory (*sv*) and chemosensory (*nompA*); and (3) a small cluster (marked by *Wnt6*, *Lim1*) that was not identified in the Fly Cell Atlas. Since the analysis robustly recovered the published benchmark, we moved on to analyzing cell types in surviving and injured limbs with developing protrusions.

Surviving tissues from injured limbs at the protrusion stage show all the major clusters of epithelial, muscle, hemocyte, and neuron cells (Fig. 6E). There are changes in the proportion of the clusters, and the chemosensory support cells are not recovered. Notably, however, there is an increase in the proportion of the muscle and epithelial cells. Importantly, the injured, surviving limbs contain a distinct muscle cluster and a distinct epithelial cluster (Fig. 6E, orange bars, and Fig. 7A), which are not normally found in the limbs (Fig. 6D). These new clusters are not small. The new muscle cluster comprises 21.3% of the total muscle cells. The new epithelial cluster comprises 43.5% of the total epithelial cells. In each cluster, the canonical fate markers (e.g. *Mhc* for muscle or *grh* for epithelial) rank lower and are replaced by different top genes (Table S2), discussed next. We verified that these distinct clusters cannot be recovered from the intact limb cells even when we overclustered (Table S2). We next analyzed these new clusters in more depth.

**Fig. 7.**
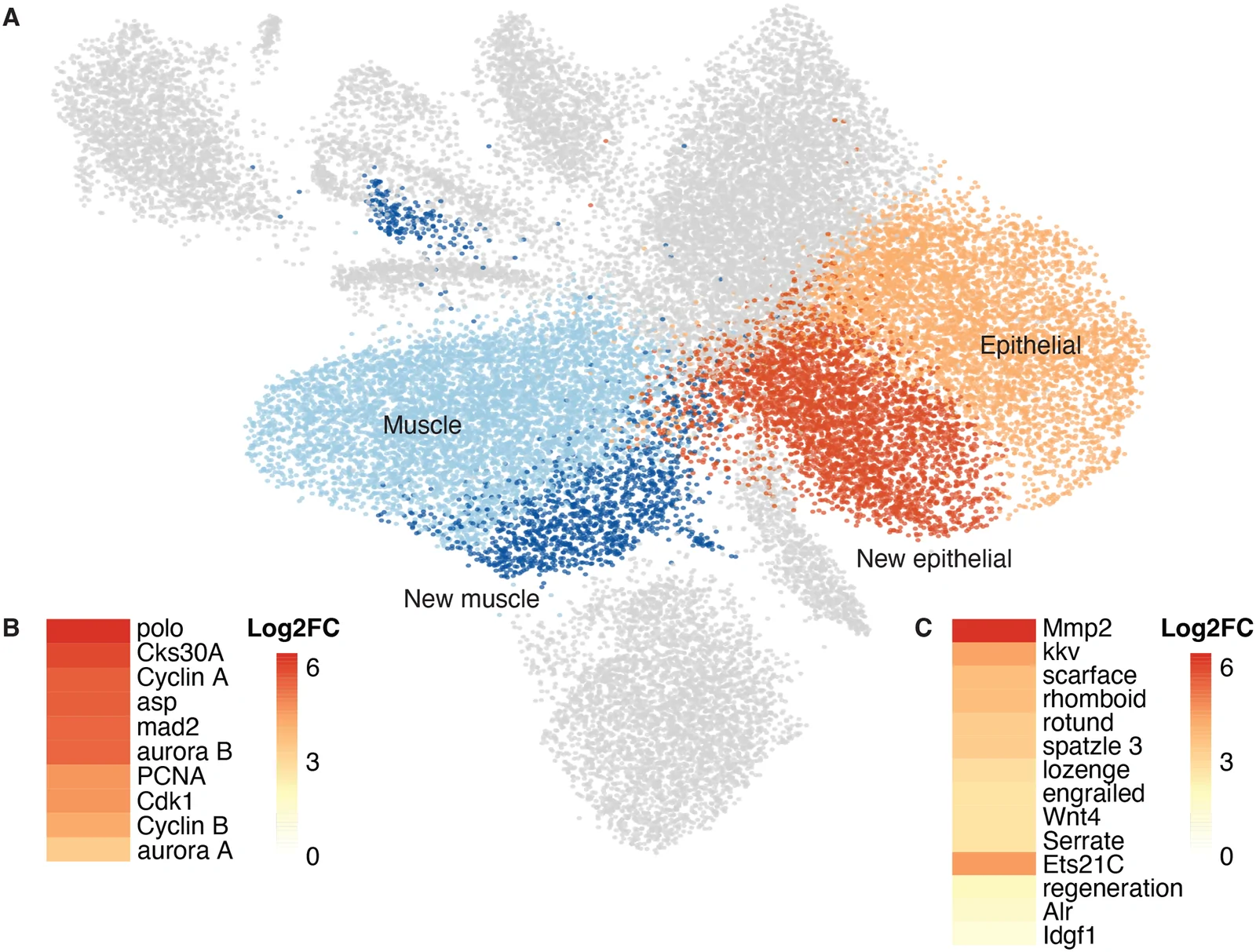
**The muscle and epithelial clusters specific to the injured, surviving limbs are enriched in mitotic and morphogenetic genes.** (A) UMAP of treated cells, highlighting the new muscle (dark blue) and new epithelial (dark orange) clusters that are specific to the injured, surviving limbs. (B,C) Heatmaps of differentially expressed genes in the new muscle cluster relative to the muscle cluster in intact limbs (B) and in the new epithelial cluster relative to the epithelial 2 cluster in intact limbs (C). Genes are grouped by representative GO categories. Color scale indicates log2 fold change. See Table S3 for the full list of differentially expressed genes and Table S4 for the full list of enriched GO terms.

### The new muscle and epithelial clusters are enriched in mitotic and morphogenetic genes

Starting with the muscle cluster, we compared gene expression in the new muscle cluster in the injured, surviving limbs with that in the muscle cluster in the intact limbs (Fig. S6, Table S3). Top upregulated genes in the new muscle clusters are dominated by multiple genes in the heat shock and antioxidant glutathione pathways (Fig. S6A). The expression of heat shock proteins in muscles has been shown to promote proteostasis and protection from stress, even conferring nonautonomous protection to surrounding cells (Kawasaki et al., 2016). Also notably enriched in the upregulated genes are the mitotic Gene Ontology (GO) terms (Fig. S6B). Of the differentially expressed genes, 11% are mitotic, including *Cyclin B*, *Cyclin A*, *Cdk1*, *PCNA*, *Cks30A*, *polo*, *aurora A*, and *aurora B* (Fig. 7B). In parallel with upregulation of the mitotic genes, interestingly, markers of differentiated muscles are significantly downregulated, including *Mhc*, *sals*, *sls*, and *Zasp52* (Fig. S6A, Table S3). Together, these results identify a distinct muscle cell state marked by cell-cycle-related transcriptional remodeling and reduced differentiation signatures. Succeeding studies will certainly benefit from a direct assessment of where proliferation takes place.

Next, we compared gene expression in the new epithelial cluster in the injured, surviving limbs with that in the corresponding epithelial cluster in intact limbs (Fig. S7, Table S3). Top upregulated genes in the new epithelial clusters are again dominated by the aforementioned heat shock proteins as well as genes in the JAK/STAT pathway. Enriched in the upregulated genes are notably GO terms in development and morphogenesis, including imaginal disc morphogenesis, post-embryonic organ development, and epithelial morphogenesis (Fig. S7; see some genes highlighted in Fig. 7C). The analysis notably recovers the GO terms of tissue regeneration. Although small, the genes driving this enrichment remarkably include, all upregulated, *regeneration* (*rgn*, by fourfold), *Augmented of liver regeneration* (*Alr*, threefold), and *imaginal disc growth factor 1* (*Idgf1*, 2.8-fold), all of which are key genes in imaginal disc regeneration (McClure et al., 2008; Katsuyama et al., 2015). Consistent with this enrichment is upregulation in *Ets21C* (24-fold), a stress-induced transcription factor recently shown to sustain a pro-regenerative transcriptional program in imaginal disc (Worley et al., 2022).

Finally, in addition to the formation of new cell clusters, the injured, surviving limbs also show marked gene expression modulation in the remaining cell types (relative to the corresponding cell types in intact limbs). Different cell types respond differently. For instance, glial (*repo*+) cells show upregulation in ribosome biogenesis, protein folding, and glial cell development. In tracheal (*trh*+) cells, top upregulated GO terms are immune response, cytoskeleton organization, and tracheal system development. To verify that the conclusions of the differential expression analysis are not sensitive to clustering parameters, we performed combined analysis, comparing all cells. This analysis recovered the key genes and GO terms, as seen in Tables S3 and S4.

In summary, the single-nucleus data characterize cell states and gene markers present in the surviving injured limbs compared to intact (untreated, uninjured) limbs. The analysis identifies the formation of large, new muscle and epithelial cell types and marked gene modulation across all cell types. The protocol developed here, together with the markers identified, can facilitate further studies to probe the individual effects of wound healing, injury, or treatment on the cell state composition.

## DISCUSSION

This study characterizes the early processes mounted by amputated adult *Drosophila* limb in response to nutrient treatment. Within hours, while limbs of the untreated flies normally heal wounds scabbed, a large fraction of limbs of the flies treated with nutrient factors show alteration in wound healing, by healing scabless. Subsequently, limbs that heal scabless show a dramatic reversal of large-scale tissue loss. Finally, in limbs with tissue survival, protrusion forms over the amputation site that is marked by formation of distinct cell states that are not normally found in limbs. These new cell states are enriched with mitotic and morphogenetic genes, including those that are known to drive regeneration in the larval tissues. These findings provide molecular evidence consistent with the partial limb regrowth suggested in previous work (Abrams et al., 2021; reproduced in Fig. 1) and more subsequent studies (Bates et al., in review, 2026; Li et al., in review, 2026; reproduced in Fig. 1). While the reported growth occurs over the month scale, the hour- and day-scale wound healing, tissue, and cell markers characterized in this study can facilitate future studies into the mechanisms underpinning the onset of regrowth.

While regrowing a complete organ may still be a long-term goal in the field and there are questions as to how far we can get there by endogenous interventions alone, there is the notion of ‘useful intermediates’. At the cell and tissue level, findings from studies of assessing how to promote regeneration processes continue to push our imagination of the extent to which cellular processes may remain remarkably plastic in the adult context. Insights at this level can already be useful for informing current therapeutic strategies. For instance, large-scale tissue loss and muscle atrophy are known complications in major injuries, e.g. in amputees or intensive care unit patients, and can slow down recovery or lead to difficulties in prosthetic use (Kress and Hall, 2014; Sanders and Fatone, 2011). The findings in this work suggest that a relatively simple intervention with specific nutrient factors may substantially reduce the extent of tissue loss.

Consistent with the role of nutrients, there are growing findings that metabolic reprogramming of the injured tissue can promote regeneration, e.g. in mouse skeletal muscle and heart (e.g. Shang et al., 2020; Li et al., 2023a; Shi et al., 2024). The role of nutrient and metabolic factors suggests that, in addition to developmental pathways, energy and/or resources might be limiting in regeneration activation. If further verified, the suggested induction of limb regrowth in adult *Drosophila* can provide a useful phenotype with which to investigate the mechanistic connection between nutrient sensing and regeneration. Interestingly, the more detailed characterization we undertook in this study reveals that a small fraction of the flies already spontaneously mount the very early processes that may facilitate growth. This suggests that the condition that promotes regrowth may not be that deeply hidden and potentially remain accessible to natural variations.

## MATERIALS AND METHODS

### Fly lines

The following fly lines were used in this study: wild-type CantonS (a gift from Kai Zinn lab at Caltech), Mef2>RFP (BDSC #26882), and NRE-GFP (BDSC #30727). The flies were kept at room temperature, with a 12-h/12-h light/dark cycle.

### Amputation

The flies were gently anesthetized with CO_2_, <3–4 l/min, which left them immobilized but still jittery, and the tibia was then cut using fine spring scissors (Fine Science Tools #91500-09). More than five flies at a time were anesthetized to keep the anesthesia time for <1 min. It should be noted that the wound healing and subsequent regrowth can be sensitive to amputation methods and these handling parameters (e.g. Sustar and Tuthill, 2023; Li et al., 2023b); thus, care should be taken to minimize stress. Amputation was performed on the midlimb and hindlimb, except for single-nucleus RNA sequencing, in which only midlimbs were used for consistency in tissue collection. After amputation, the flies were placed in treated food (see ‘Treatment’) at a rate of four to six flies per vial. Limbs were scored as scabbed if a complete melanized scab covered the wound site; all other limbs, including those with light or partial melanization, were scored as scabless.

### Treatment

To supplement the food with leucine, glutamine, and insulin (LGI), we prepared a stock LGI solution at 1.7 mM L-leucine (Sigma-Aldrich L8912), 1.7 mM L-glutamine (Sigma-Aldrich G8540), and 0.03 mg/ml insulin (Sigma-Aldrich I0908). The fly food was briefly microwaved to melt the top layer, and then 200 μl of the LGI solution was mixed into the melted top layer. To supplement the food with yeast, dried yeast granules (Saf-Instant Yeast) were added to cover 30–40% of the food area. The treatment protocol is described in Fig. 2B. The duration, dose, and combination of molecules were previously tested by Abrams et al. (2021); the protocol used here gives the strongest response so far.

### Imaging

Brightfield imaging was performed using a Zeiss Axio V16 dissection microscope and an Axiocam 208 color camera. For confocal fluorescence imaging, the limbs were dissected and then mounted in a small drop of Elmer's clear glue (Koyama et al., 2020). A #1.5 cover glass was gently pressed down onto the limb. The mounted limbs were then imaged immediately with a Zeiss LSM 980 using a Zeiss Plan-Apochromat 63×/1.40 Oil DIC M27 oil-immersion objective. Mef2>RFP was imaged with a 10% 561 nm laser and 750 V photomultiplier tube (PMT) gain. NRE-EGFP was imaged with a 10% 488 nm laser and 750 V PMT gain. For both lines, images were acquired using a scan speed of 6, a frame size of 1024×1024 pixels, 4× averaging, and an 8 bit pixel depth. Within a figure, images are shown with an identical dynamic range for consistent comparison.

### Single-fly tracking

For single-fly tracking, the flies were amputated one at a time and housed individually, with one fly per vial, so that each fly could be identified and followed across imaging time points. Flies were amputated as described above. CO_2_ exposure for each fly was kept under 30 s to minimize stress. The flies were imaged immediately after amputation (0 dpa) and again at 4–6 weeks after amputation. To steady the flies, a customized chamber was made by placing four #1.5 coverslips as spacers on each end of a clear glass slide. The fly was placed under the chamber, and a snapshot was taken with a Zeiss Discovery V8 stereomicroscope and an Axiocam 208 color camera. Measurement of the tibia length was performed in Fiji. The length of the tibia is measured as the diameter of the smallest circle that encapsulates the tibia. The limbs were aligned using the joint boundaries as morphological markers.

### Single-nucleus isolation

Female flies were used. The treated limbs were collected at 7–10 days after amputation, selecting for those with protrusion. Control limbs were collected from the same region of limbs from uninjured, untreated flies. For each replicate, ∼1500 flies were amputated to enable collection of ∼100 limbs with the desired morphological stage within the collection time window. Dissection was performed according to the Fly Cell Atlas (Li et al., 2022). Samples were homogenized with a Dounce homogenizer. The homogenized samples were sequentially filtered through 40, 10 and 5 μm cell strainers. In each filtering step, 300 μl of homogenization buffer was added to rinse the strainer, followed by centrifugation at 300 ***g*** at 4°C for 30 s. Then, the final solution was centrifuged at 300 ***g*** for 10 min at 4°C to pellet the nuclei. Approximately 30,000 nuclei per sample were loaded into 10x Genomics Chromium X for droplet generation. Libraries were prepared using the 10x Genomics Chromium GEM-X Single Cell 3′ v4 kit and sequenced on a NovaSeq X Plus with a 25B flow cell at the University of California, San Francisco Center for Advanced Technology (UCSF CAT).

### Single-nucleus RNA sequencing analysis

The raw sequencing reads were demultiplexed by UCSF CAT. The Kallisto reference genome index was built with kb-python (v0.29.5) using the *D. melanogaster* BDGP6.54 genome FASTA and corresponding Gene Transfer Format (GTF) annotation. Alignment and quantification were performed using kallisto (v0.51.1, Bray et al., 2016; Melsted et al., 2021; Sullivan et al., 2025). The count matrices were imported into Python (v3.9.5) and analyzed using Scanpy (v1.10.3; Wolf et al., 2018). Cells were filtered using the stringent threshold of the Fly Cell Atlas; i.e. cells were required to have a minimum of 500 UMI, 200 genes, and <5% mitochondrial content. Genes were required to be present in at least three cells to be included in the analysis. Cells with a Scrublet doublet score >0.3 were removed (Wolock et al., 2019). The filtered datasets were concatenated into control and treated groups and normalized to 10^6^ counts and log1p-transformed. Highly variable genes were calculated using the Seurat method with sc.pp.highly_variable_genes (min_mean=0.01, max_mean=8, min_disp=1, n_bins=20, flavor=’seurat’; Satija et al., 2015). PCA at the cell level was performed using sc.tl.pca (svd_solver=arpack). PCA at the sample level was performed by summing raw counts within each biological replicate to generate sample-level expression profiles, followed by normalization, log1p transformation, and PCA using the sklearn PCA implementation. A neighborhood analysis was performed using sc.pp.neighbors (*n*_neighbors=30, *n*_pcs=20). Dimensionality reduction for visualization was performed using Uniform Manifold Approximation and Projection (UMAP), which is implemented in Scanpy (Becht et al., 2019). Clustering was performed using the Leiden algorithm (Traag et al., 2019; see Table S2). We identified a small cluster in control that consisted predominantly of cells from replicate 4, which was further verified using the CellSweep algorithm (Caskey et al., 2026 preprint) and was found to contain only ambient RNAs and was therefore excluded from downstream analysis. Cluster-specific top marker genes were computed using sc.tl.rank_gene_groups (method=Wilcoxon). Differential gene expression analysis was performed using DESeq2 (v1.34.0; Love et al., 2014). GO enrichment analysis was performed using *clusterProfiler* (v4.2.2; Yu et al., 2012) with the gene background defined as all genes expressed in the analyzed cells. GO term similarity clustering was performed using rrvgo (v1.6.0; Sayols, 2023).

## Supplementary Material

10.1242/biolopen.062737_sup1 Supplementary information

Table S2. Clustering analysis and top ranked gene list.Ta

Table S3. Table of differentially expressed genes across cell types.

Table S4. Gene Ontology enrichment analysis across cell types.
