# Supplementary material for: Early injury responses to nutrient treatment in adult Drosophila limb
Source: Biol Open. 2026 Oct 23;15(10):bio062737. doi: 10.1242/bio.062737 (PMC13730125; doi:10.1242/bio.062737)
Supplement: Supplementary file 1 [file biolopen-15-062737-s1.pdf]

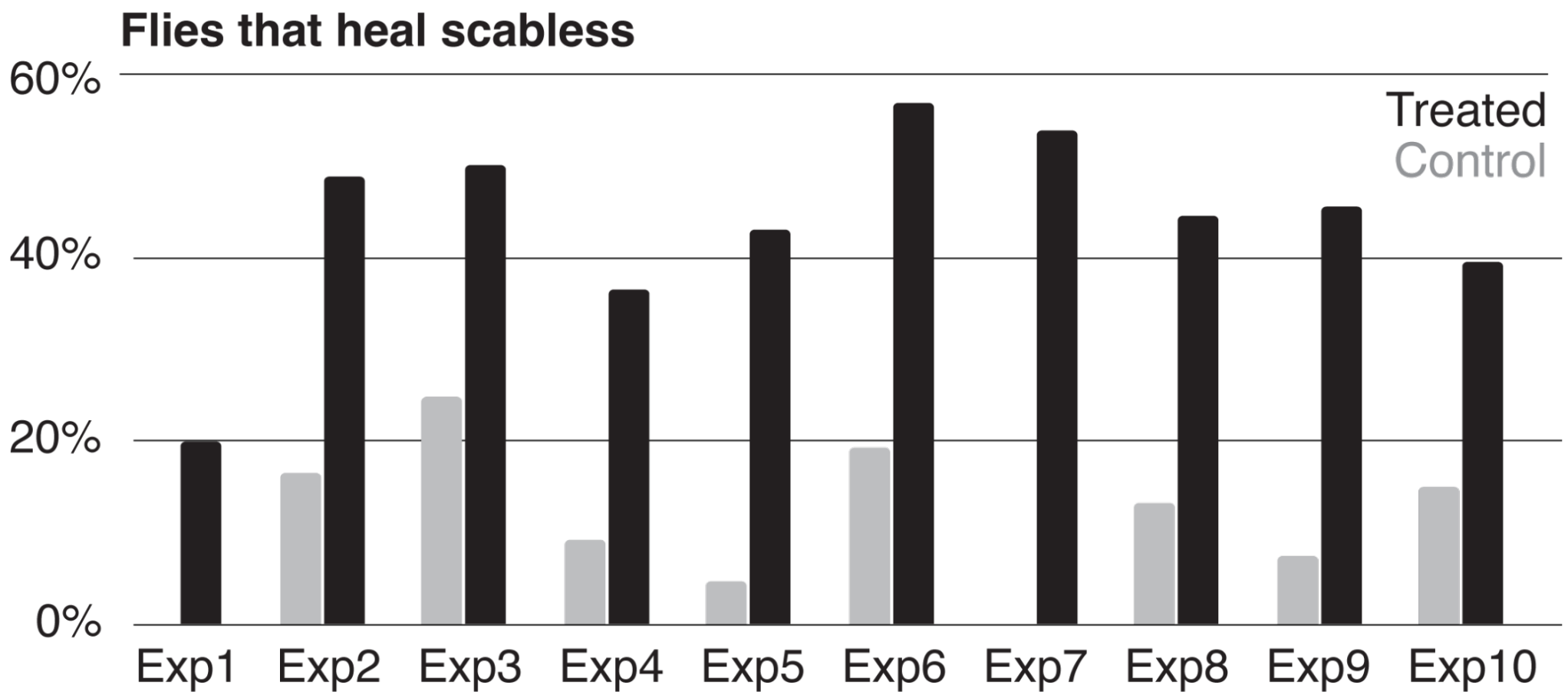

Estimate fold change	95% CI	p-value
3.4	2.4, 4.8	<0.0001 ***

Fig. S1. Despite variation, the nutrient treatment reproducibly increases the percentage of flies that heal wounds scabless.

These experiments were performed in the wild-type CantonS flies. Shown here are ten independent experiments, in which control (grey) and treated (black) conditions were set up side-by-side. Independent experiments are experiments performed using different fly cohorts in different weeks (a fly cohort are flies that emerge in the same stock bottle, i.e., from the same set of parentals). For Exp1 and Exp7, the grey bar is missing because the scabless frequency in control is 0. Statistical significance and reproducibility were assessed by analyzing the effect size across experiments using meta-analysis (Borenstein et al., 2009), implemented using the metafor package in R (Viechtbauer, 2010), with fixed-effect model and statistical coefficients based on normal distribution. The effect size of the treatment was computed as the fold change between the scabless frequency in treated and control within an experiment (the metric risk ratio RR in Borenstein et al., 2009). Fold change = 1 means the treatment has no effect. A treatment is reproducible if the 95% confidence intervals (95% CI) of the effect size exclude 1. The p-value evaluates the null hypothesis that the fold change is 1.

Biology Open • Supplementary information

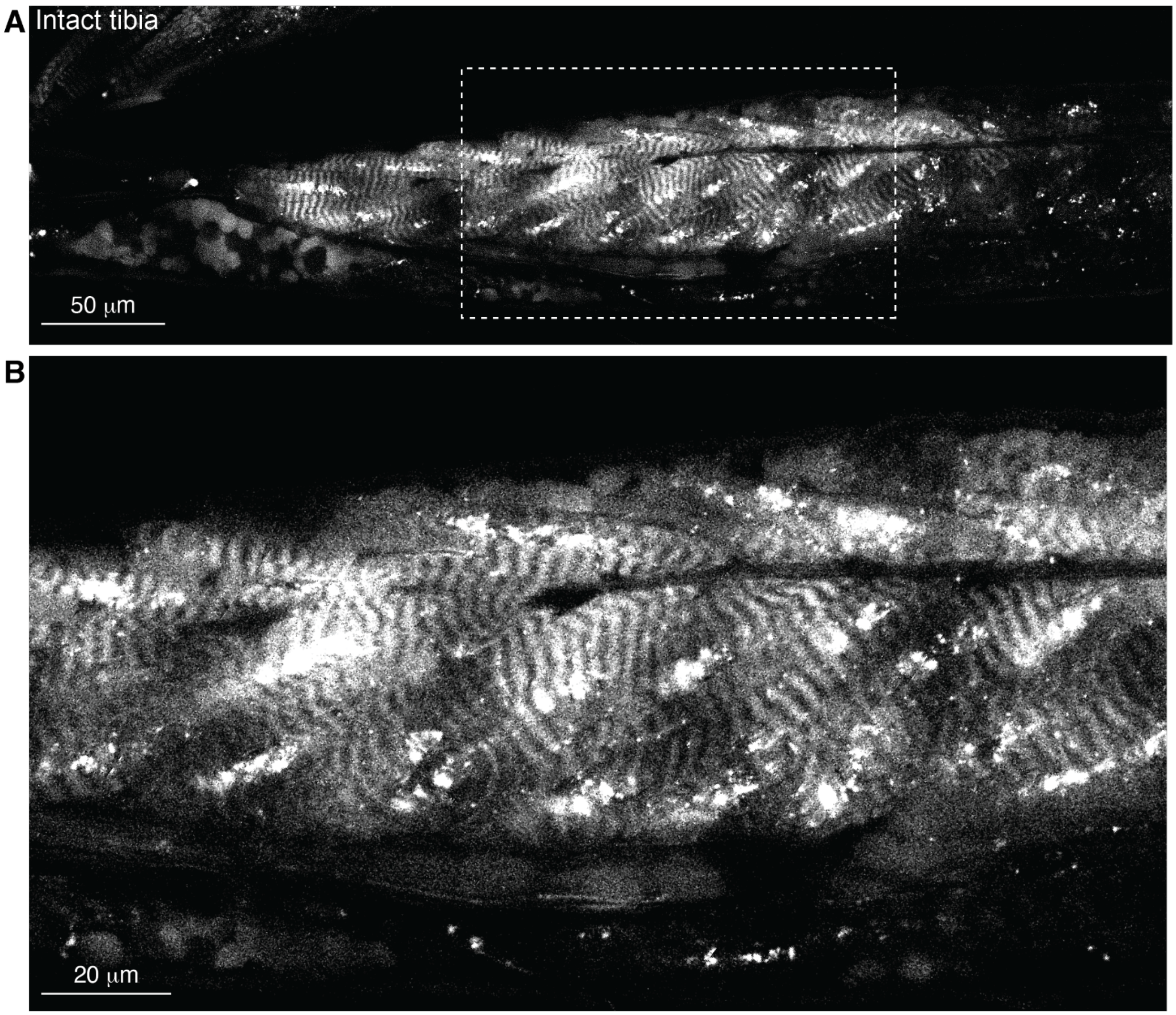

Fig. S2. Mef2>RFP expression in the unamputated adult limb.

(A) Representative image of an unamputated Mef2>RFP limb showing fluorescence enriched in striated muscle bundles. **(B)** Magnification of the boxed region in **(A)**. To image, whole limbs were dissected from Mef2>RFP flies, mounted, and imaged using Zeiss LSM 980 confocal microscope (see Methods).

Biology Open • Supplementary information

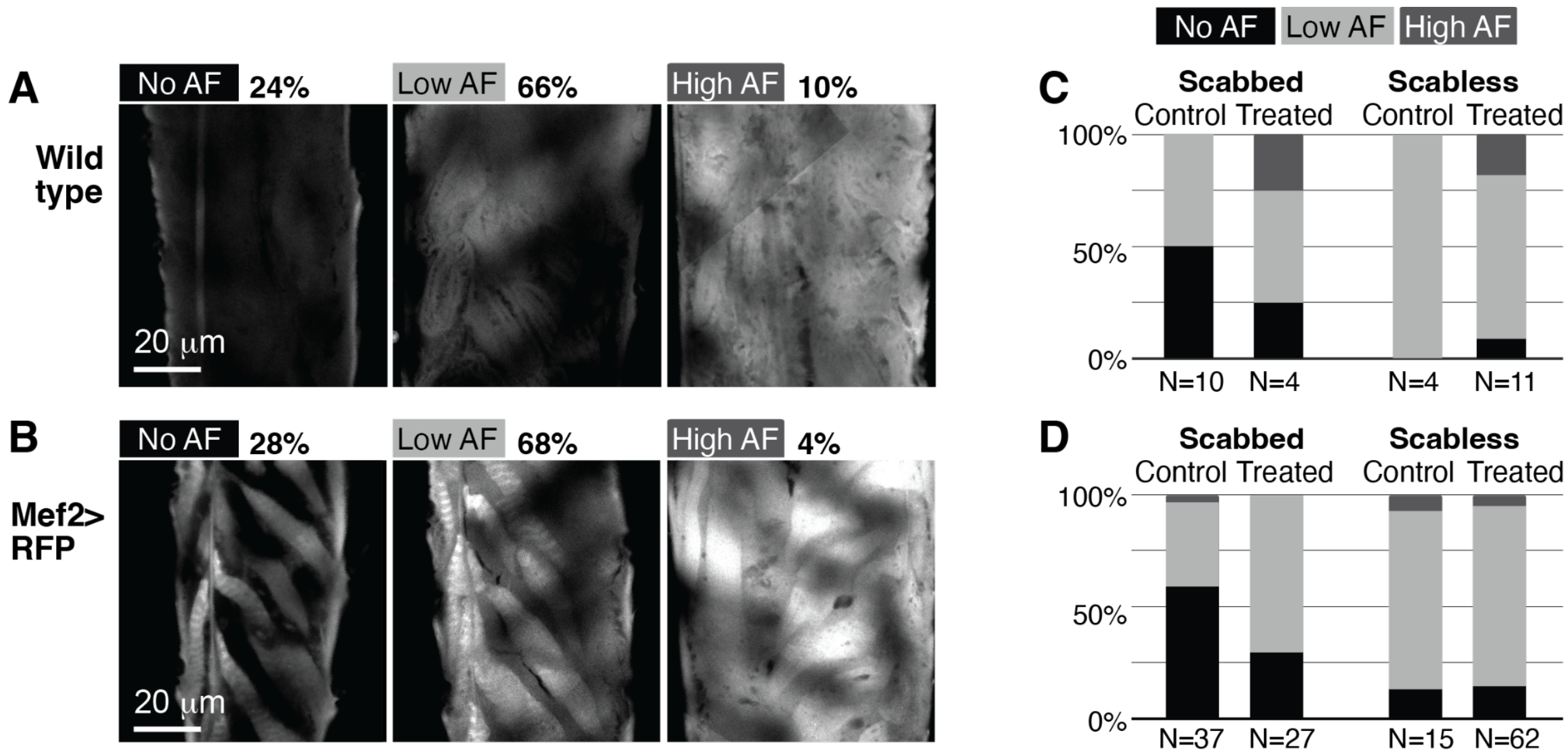

Fig. S3. Analysis of autofluorescence in injured limb segments.

Autofluorescence (AF) is a common marker of increased metabolic activities. Major sources of endogenous tissue AF are high-energy molecules, such as NADPH and flavins (Monici, 2005; Morrow et al., 2024). **(A, C)** To assess the extent of AF, we examined wild-type fly limbs at 4-14 dpa using imaging parameters identical to those used to analyze Mef2>RFP. AF categories were scored visually based on overall signal intensity: no visible AF, low diffuse AF, or strong AF with near-saturated signal. Since the wild-type flies carry no exogenous fluorescent constructs, all signals observed can be attributed to endogenous AF. Low AF is detected in 66% of the limbs analyzed. AF is confined to the injured segment; no or low AF signals were observed in the femur. **(B, D)** Mef>RFP signals can be readily distinguished from the low AF. While the AF signal is diffuse with some weak tissue outlines **(A)**, Mef2>RFP signals are higher in intensity and show a sharp pattern of muscle bundles with striation **(B)**. In addition, we also find in 4-10% of the limbs analyzed signs of unhealthy tissues, e.g., delamination from cuticle, holes in tissues, which correlates with very high autofluorescence (high AF). These limbs were excluded from the analysis.

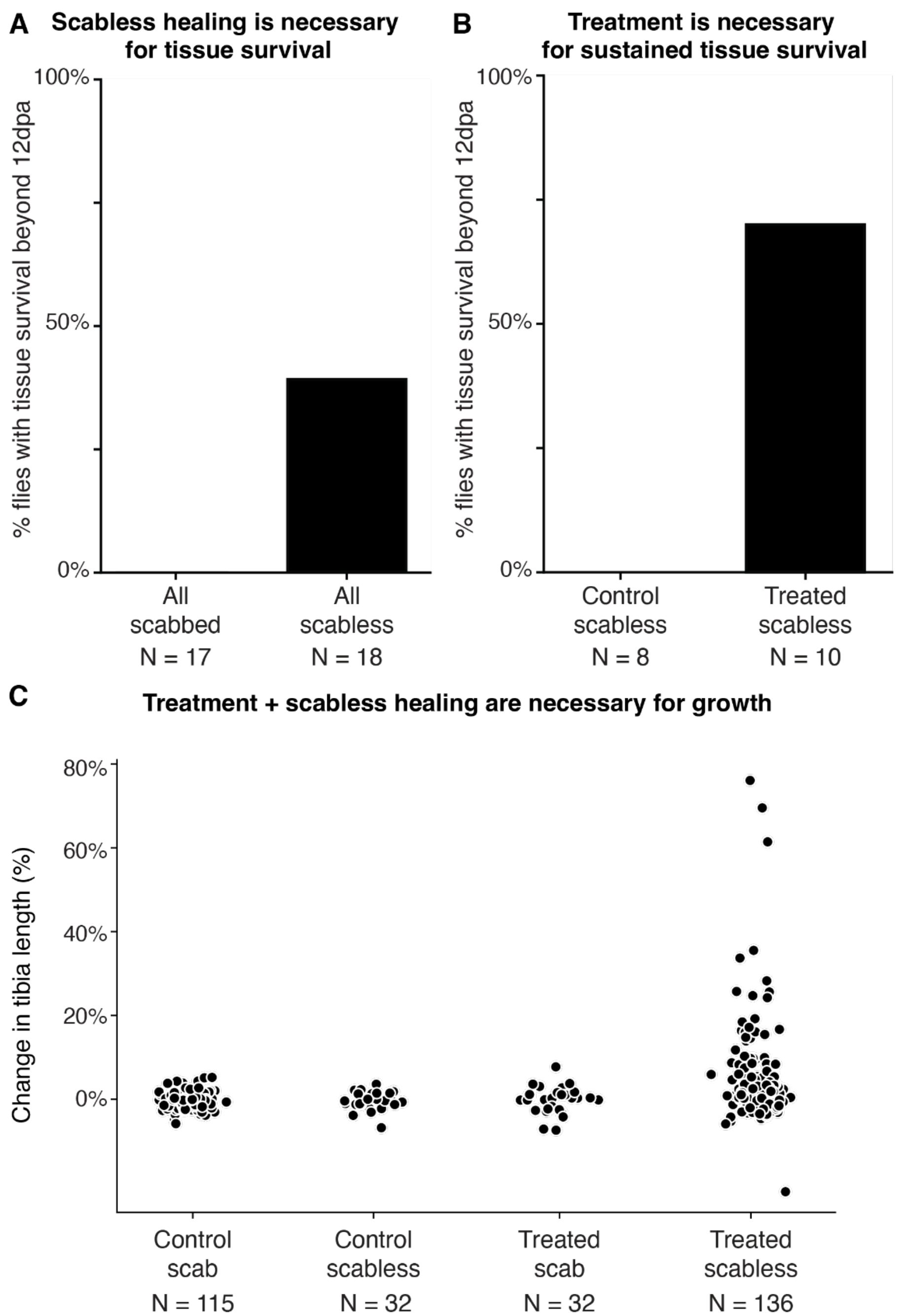

Fig. S4. Scabless healing is necessary for tissue survival and regrowth.

(A–B) Percentage of flies with full Mef2>RFP signal in amputated limbs beyond 12 dpa. **(A)** Across all conditions, control and treated, scabbed limbs showed no detectable Mef2>RFP signal, whereas 38.9% scabless limbs retained detectable tissue survival. **(B)** Among scabless limbs, sustained full tissue signals were observed only in treated flies.

(C) Change in tibia length at 4 weeks post amputation. To measure tibia growth, flies were imaged immediately after amputation and then imaged again at 4 weeks post amputation. This plot comes from reanalysis of the dataset in Abrams et al. (2021) and newly collected data. Regrowth is only observed in treated scabless limbs.

Biology Open • Supplementary information

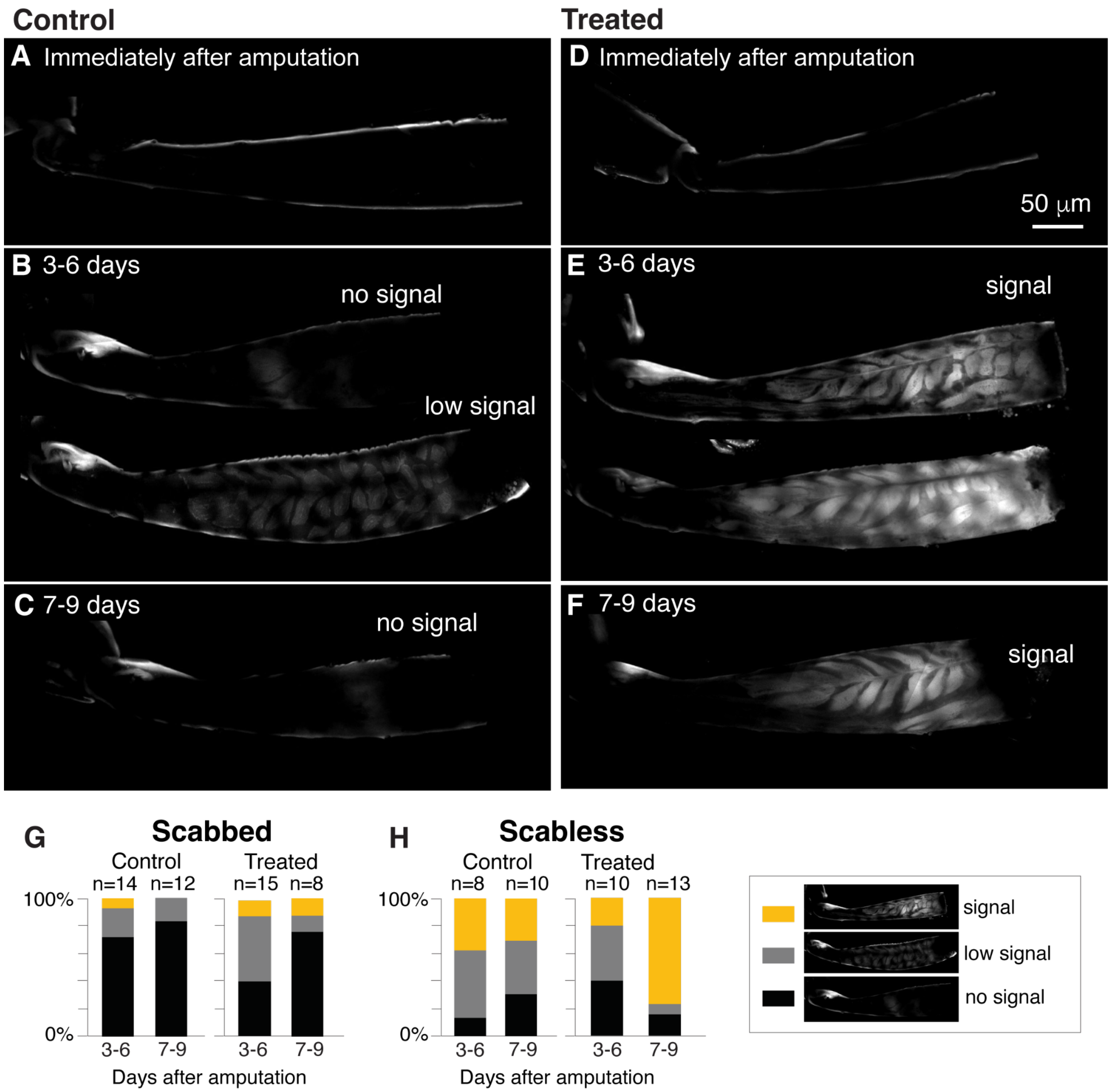

Fig. S5. Notch reporter is activated in the treated, scabless limbs.

Notch activation in the muscle bundle is required for muscle repair; in aged and muscular dystrophic mice, Notch activation improves repair myogenesis (Bi et al., 2016). To assess Notch signaling, we used the established reporter for Notch signaling, NRE-EGFP, in which EGFP expression is driven by the Notch Response Elements (NRE; Zacharioudak and Bray, 2014). Time in the images is days after amputation. Each image is a representative tibia from an independent fly at the indicated time point. NRE-EGFP is not active in the intact limbs or upon amputation **(A**, **D)**, and remains inactive or low in limbs that heal scabbed (**B–C**, **G**). NRE-GFP is strongly activated in treated, scabless limbs (**E**, **F**, **H**).

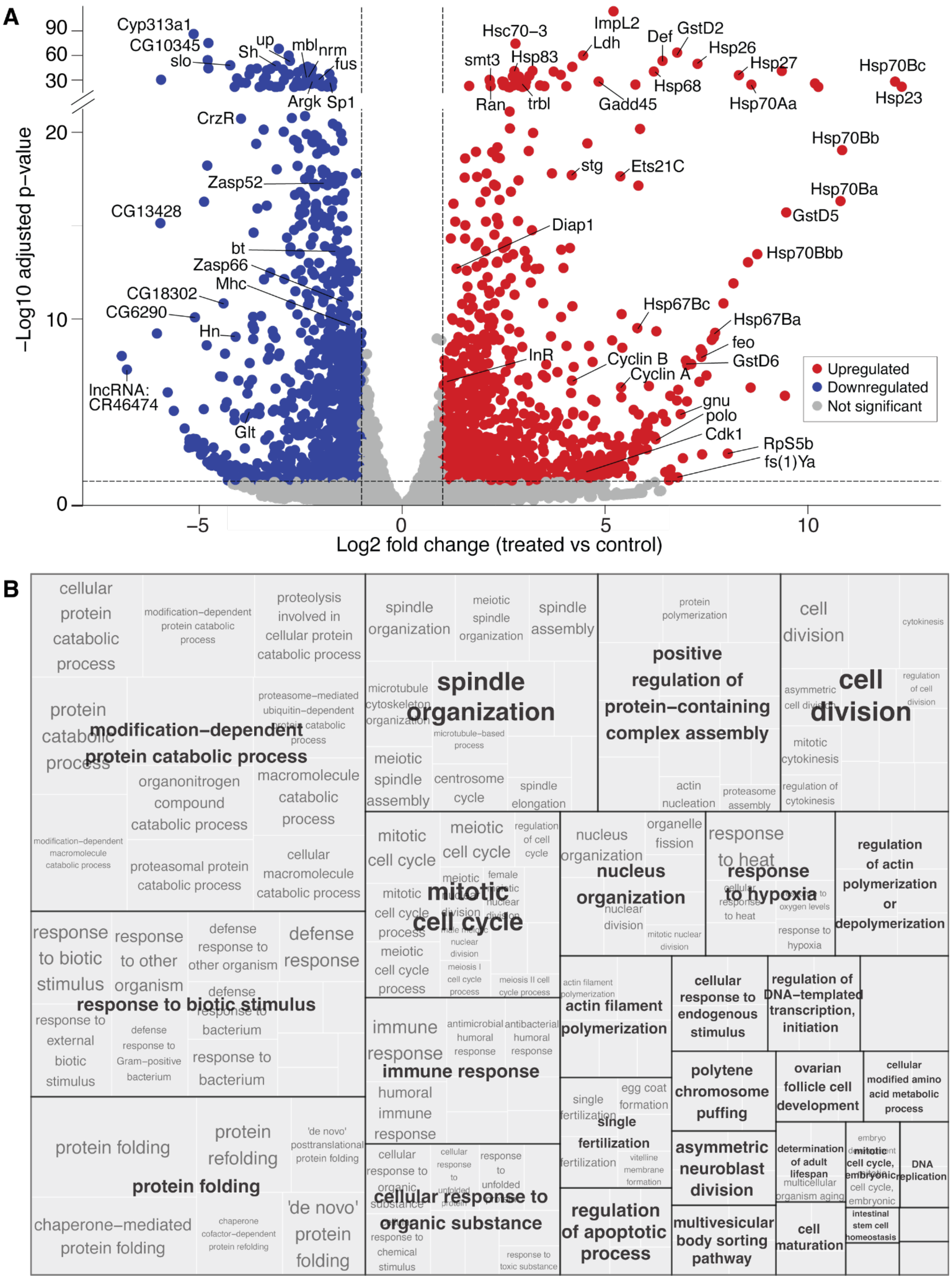

Fig. S6. Genes differentially expressed in the new muscle cell cluster.

Differential expression (DE) analysis was performed between the newly clustered muscle cells in the limbs with protrusion and the muscle cluster in the normal limbs. **(A)** Volcano plot of DE genes with cut-off adjusted p-value > 0.05, fold change > 1. **(B)** GO terms enriched in the DE genes.

Biology Open • Supplementary information

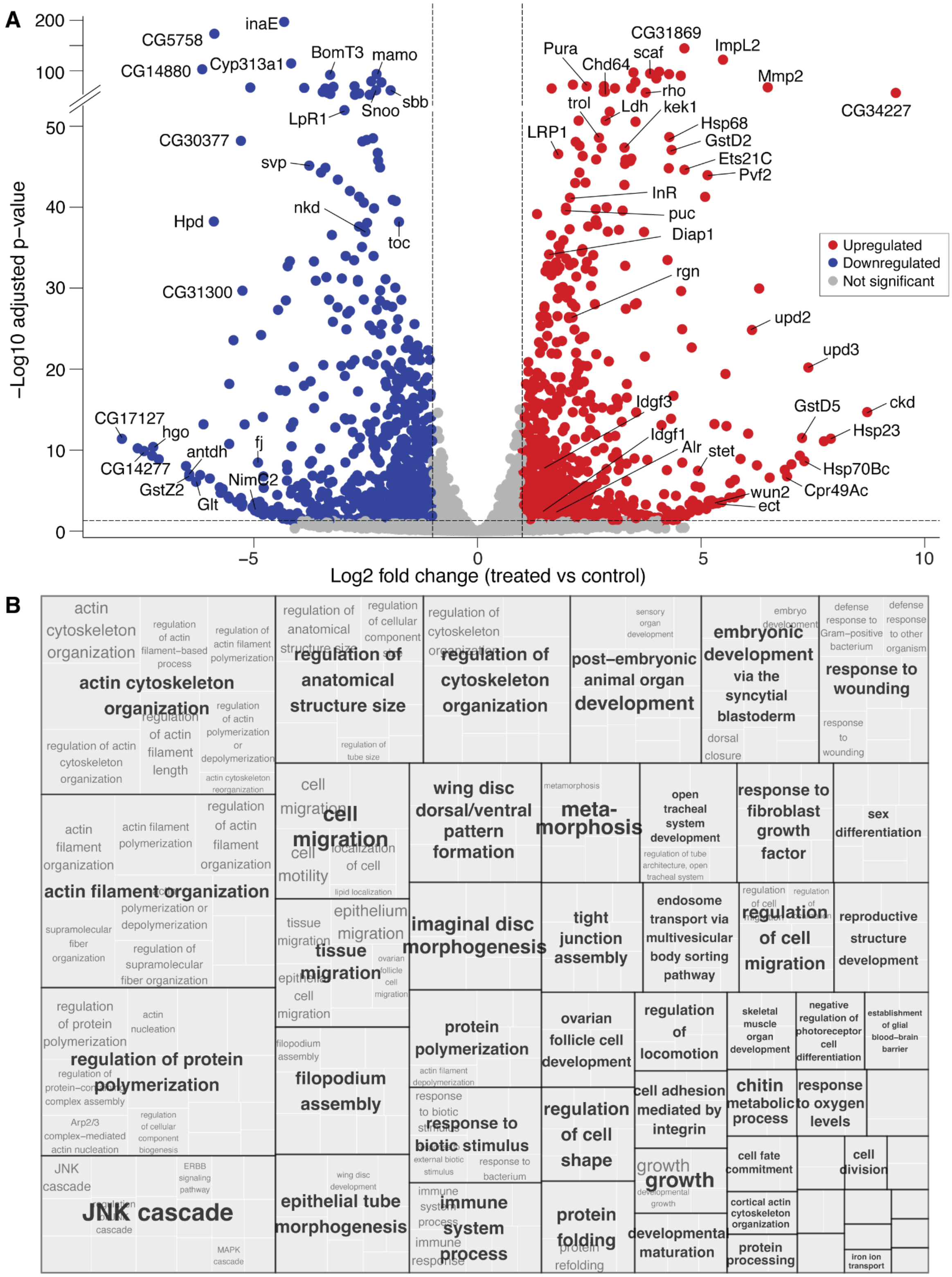

Fig. S7. Genes differentially expressed in the new epithelial cell cluster.

Differential expression (DE) analysis was performed between the newly clustered epithelial cells in the limbs with protrusion and the corresponding epithelial cluster in the normal limbs. **(A)** Volcano plot of DE genes with cut-off adjusted p-value > 0.05, fold change > 1. **(B)** GO terms enriched in the DE genes.

Table S1. Wound healing and response to treatment are influenced by genotype.
The genotype of a reporter line differs from wild type because of the inclusion of the reporter construct itself, as well as the balancer chromosomes and marker mutations. To use a reporter line, we first verified that the line can recapitulate the wild-type in responding to treatment. Shown are the averages. N is the total number of flies examined. Some reporter lines do not respond to treatment (e.g., ubi-FUCCI, nls-GFP) or appear to have a wound healing phenotype (e.g., puc- GFP), and therefore could not be used for this study. BDSC: Bloomington *Drosophila* Stock Center.

**Fly line**	**BDSC stock number**	**Baseline rate of scabless healing**	**Rate of scabless healing with treatment**
Some lines that respond to treatment			
Mef2>mRFP	26882	9% (N=266)	31% (N=405)
NRE-EGFP	30727	21% (34)	66% (N=40)
His2B-RFP	Gift from David Glover lab	12% (N=189)	35% (N=272)
Some lines that did not significantly respond to treatment			
Zasp-GFP	6824	6% (N=34)	13% (N=103)
nls-GFP	1691	13% (N=16)	6% (N=33)
A line with a high baseline of scabless wound healing, and did not significantly respond to treatment			
puc-GFP	56276	60% (N=45)	60% (N=10)

Table S2. Clustering analysis and top ranked gene list.Ta

Available for download at
https://journals.biologists.com/bio/article-lookup/doi/10.1242/bio.062737#supplementary-data

Table S3. Table of differentially expressed genes across cell types.

Available for download at
https://journals.biologists.com/bio/article-lookup/doi/10.1242/bio.062737#supplementary-data

Table S4. Gene Ontology enrichment analysis across cell types.

Available for download at
https://journals.biologists.com/bio/article-lookup/doi/10.1242/bio.062737#supplementary-data

Supplement References

Bi P, Yue F, Sato Y, Wirbisky S, Liu W, Shan T, Wen Y, Zhou D, Freeman J, Kuang S. Stage-specific effects of Notch activation during skeletal myogenesis. Elife. 2016 Sep 19;5:e17355. doi: 10.7554/eLife.17355. PMID: 27644105; PMCID: PMC5070950.

Borenstein M Hedges LV Higgins JPT Rothstein HR. Introduction to Meta-Analysis Chichester, 2009 UK: John Wiley & Sons, Ltd.

Monici M. Cell and tissue autofluorescence research and diagnostic applications. Biotechnol Annu Rev. 2005;11:227-56. doi: 10.1016/S1387-2656(05)11007-2.

Morrow CS, Tweed K, Farhadova S, Walsh AJ, Lear BP, Roopra A, Risgaard RD, Klosa PC, Arndt ZP, Peterson ER, Chi MM, Harris AG, Skala MC, Moore DL. Autofluorescence is a biomarker of neural stem cell activation state. Cell Stem Cell. 2024 Apr 4;31(4):570-581.e7. doi: 10.1016/j.stem.2024.02.011. Viechtbauer W. Conducting Meta-Analyses in R with the metafor Package. J. Stat. Soft. 2010 36:e3. doi: 10.18637/jss.v036.i03.

Zacharioudaki E, Bray SJ. Tools and methods for studying Notch signaling in *Drosophila melanogaster*. Methods. 2014 Jun 15;68(1):173-82. doi: 10.1016/j.ymeth.2014.03.029.
